# Sensitive capacitive pressure sensors based on graphene membrane arrays

**DOI:** 10.1038/s41378-020-00212-3

**Published:** 2020-11-16

**Authors:** Makars Šiškins, Martin Lee, Dominique Wehenkel, Richard van Rijn, Tijmen W. de Jong, Johannes R. Renshof, Berend C. Hopman, Willemijn S. J. M. Peters, Dejan Davidovikj, Herre S. J. van der Zant, Peter G. Steeneken

**Affiliations:** 1 Kavli Institute of Nanoscience, Delft University of Technology, Lorentzweg 1, 2628 CJ Delft, The Netherlands; 2 Applied Nanolayers B.V., Feldmannweg 17, 2628 CT Delft, The Netherlands; 3 Department of Precision and Microsystems Engineering, Delft University of Technology, Mekelweg 2, 2628 CD Delft, The Netherlands

**Keywords:** Nanosensors, NEMS, Sensors

## Abstract

The high flexibility, impermeability and strength of graphene membranes are key properties that can enable the next generation of nanomechanical sensors. However, for capacitive pressure sensors, the sensitivity offered by a single suspended graphene membrane is too small to compete with commercial sensors. Here, we realize highly sensitive capacitive pressure sensors consisting of arrays of nearly ten thousand small, freestanding double-layer graphene membranes. We fabricate large arrays of small-diameter membranes using a procedure that maintains the superior material and mechanical properties of graphene, even after high-temperature annealing. These sensors are readout using a low-cost battery-powered circuit board, with a responsivity of up to $47.8$ aF Pa^−1^ mm^−2^, thereby outperforming the commercial sensors.

## Sensors that perform well under pressure

Arrays of tiny graphene membranes exhibit excellent performance as pressure sensors, offering a competitive, low-cost alternative to commercially available systems. Graphene offers a durable and resilient material for sensors that generate a capacitive readout to pressure changes, but individual membranes are not sufficiently sensitive. Researchers led by Makars Šiškins and Peter Steeneken of the Delft University of Technology in the Netherlands have devised a strategy for fabricating graphene sensor arrays that overcome this limitation. Their method yields millimeter-scale assemblies of 10,000 double-layered graphene membranes, which can in turn be connected to an inexpensive battery-powered circuit board. The authors demonstrate that this system can outperform a state-of-the-art commercial pressure sensor, and propose that further improvements to the design and fabrication of these arrays could improve their responsiveness by a full order of magnitude.

## Introduction

Graphene grown by chemical vapor deposition (CVD) is a strong candidate for realizing next-generation sensor devices^1^. Its hermeticity^2–4^ and superior mechanical^5–7^ and electrical^8^ properties have enabled various types of gas pressure sensors. In most conventional pressure sensors, the pressure is determined from the deflection of a membrane due to the pressure difference between ambient gas and gas in a hermetically sealed reference cavity. There are different ways to readout the deflection of the membrane and thereby quantify the pressure difference. In capacitive graphene pressure sensors, the deflection is readout by measuring the capacitance between the graphene membrane and a reference electrode^9–12^. As the pressure-induced deflection increases the mechanical stress and tension in the membrane, it can be measured using the piezoresistive effect^13–15^ and can be probed via the mechanical resonance frequency^2,3,16^. In contrast, graphene squeeze-film pressure sensors^17^ and Pirani pressure sensors^18^ do not require a hermetic reference cavity and operate at small deflection, which can be beneficial for their operation range.

Resonant sensors are conceptually attractive because they potentially offer both gas sensing^19,20^ and pressure sensing^2,17^ functionality within a single device. However, the accurate readout of resonance frequencies with low-power electronics is challenging, requires the elimination of mass loading and cannot be easily scaled up to many devices in parallel. In piezoresistive and Pirani sensors, it is a challenge to eliminate nonpressure-related effects of the surrounding gas on the graphene resistance. In contrast, capacitive pressure sensors have the advantage that the membrane capacitance is rather insensitive to gas-induced changes in its mass and electrical resistance and thus depends mainly on the geometry and membrane deflection. However, it was found that a single graphene membrane with a diameter of 5 $\mu$m has too small of a responsivity ($< 0.1$ aF Pa^−1^) to be competitive with commercial sensors^12^.

In this work, we counter this drawback by creating arrays with a large number of membranes connected in parallel to increase the responsivity^12^. We present few-atom-thick pressure sensors that can compete with commercial capacitive pressure sensors using arrays of nearly 10,000 double-layer graphene (DLG) membranes. We optimize the design of the sensor elements, the chip layout and the readout electronics to attain a handheld, low cost, battery-powered electrical readout circuit capable of detecting pressure changes via the static deflection of graphene nanodrums.

## Materials and methods

### Chip design and graphene transfer

**Fig. 1:**
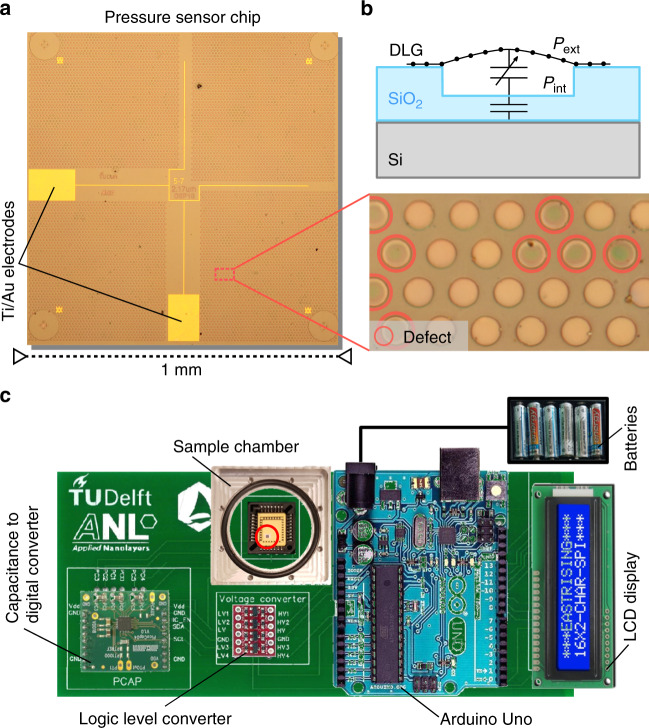
Pressure sensor and readout circuitry. **a** Optical image of the sensor chip with 10,000 circular holes, a DLG/PMMA membrane and Ti/Au electrodes. The close-up image shows the difference in contrast between intact and defect drums, with red circles indicating collapsed membranes. **b** Schematic device cross-section and capacitive pressure readout principle. **c** Readout circuitry PCB board, with the elements labeled. The total size of the PCB board is $6.9 \,\times 17.5$ cm^2^. The red circle indicates the pressure sensor chip

Simulations^12^ have shown that to achieve commercially competitive sensitivity, an array of ~10,000 circular graphene drums is needed, each with a diameter of $5$ microns. When the drums are placed on a hexagonal grid with a pitch of 10 microns between their centers, they fit on a $1 \times 1$ mm^2^ chip, as shown in Fig. 1a. To fabricate this design, Ti/Au electrodes ($5$ nm/$60$ nm), for contacting the graphene top electrode, are patterned on a silicon chip with a $285$ nm SiO_2_ layer. Then, the pattern of circular holes with a depth of $240$ nm is reactive ion etched into the SiO_2_. As shown in the cross-section in Fig. 1b, the cavity depth of the holes is less than the SiO_2_ layer thickness to prevent the graphene from touching the silicon bottom electrode, which would create an electrical short-circuit between the electrodes if one of the membranes collapses. As a last step, the graphene is transferred over the cavities. We use two layers of graphene and a small membrane diameter to improve the yield^21^ and the mechanical strength^7,22^. Since the probability that $2$ pore defects align is low, the impermeability of DLG is also much higher than that of single-layer graphene^3^.

To fabricate DLG, two sheets of CVD graphene are synthesized and then stacked on top of each other, maintaining a pristine quality interface between the sheets. Using polymethyl methacrylate (PMMA) as a support layer of $800$ nm thickness, DLG is suspended over the prepatterned circular holes in the SiO_2_/Si chip with Ti/Au electrodes (Fig. 1a). The DLG was produced and transferred to ambient pressure by Applied Nanolayers. From the differences in contrast between suspended, broken and collapsed drums^21,23^ we estimate that the dry transfer technique results in a yield of $95\!-\!99{\mathrm{\% }}$ of freely suspended DLG/PMMA membranes. The red circles in the inset of Fig. 1a indicate defect drums in a damaged region of the sample and show this difference in contrast.

### Sensor readout circuit board

The graphene capacitive pressure sensor responds to the difference established between the internal pressure of the reference cavity, $P_{{\mathrm{int}}}$, and the external pressure of the environment, $P_{{\mathrm{ext}}}$. This pressure difference results in a deflection of the atomically thin membrane and a corresponding change in capacitance^12^ of the drum, ${\mathrm{{\Delta}}}C$, as schematically depicted in Fig. 1b. We wedge-bond a $25$ $\mu$m AlSi $1{\mathrm{\% }}$ wire to Ti/Au pads on a chip for electrical contact with graphene and use a conductive silver paste to contact the Si substrate. The response of the graphene capacitive pressure sensor is high enough to be readout by chip-scale commercial electronic components, as demonstrated using the battery-powered circuit shown in Fig. 1c. Here, the sample under test is kept in a small on-board vacuum chamber that allows local control over the external gas pressure while preventing the pressure from affecting the electrical readout elements. A commercial capacitance to digital converter (AMS PCap04) is then used to record and digitize the capacitance of the sample measured at a peak-to-peak voltage $V_{{\mathrm{pp}}} = 1.5$ V with a hundred charge and discharge cycles of $5.73$ ms each. After voltage level adjustment by a logic level converter, an Arduino Uno board converts the measured capacitance into pressure using a predetermined calibration curve and displays it on an LCD screen. The circuit board is capable of measuring a change in the chip’s capacitance down to $\sim$10 aF on top of a background capacitance of a few tens of picofarads.

### Effect of PMMA removal

**Fig. 2:**
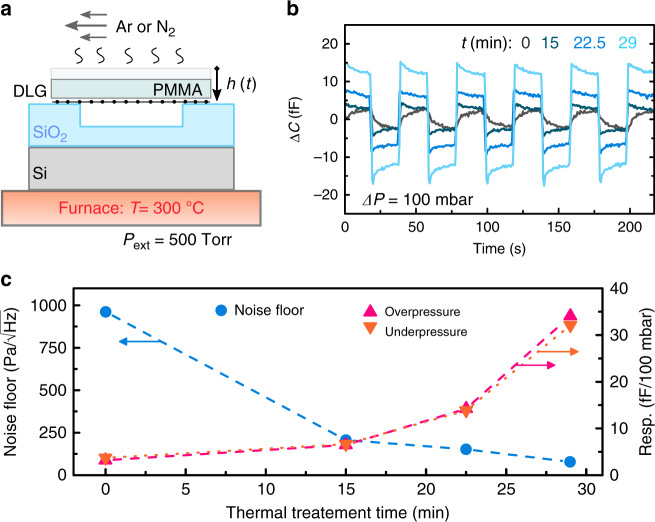
Thermal removal of the polymer and its effect on the capacitive response of the sensor. **a** Schematic explanation of the thermal annealing principle applied to reduce the PMMA thickness $h(t)$. **b** Change in capacitance of a single chip as a function of measurement in response to time-dependent pressure changes, ${\mathrm{{\Delta}}}P$, for samples that have been annealed for different times $t$ (line colors correspond to the color of the corresponding annealing times indicated in the legend). The gas pressure is changed at a rate of $175.4$ mbar s^−1^. **c** Responsivity and noise floor extracted from the data in **b**, calculated using Eq. (1)

After chip fabrication and transfer, the PMMA transfer polymer still covers the DLG sheets. In a number of previous studies, protective polymer layers were used to support graphene to increase the yield, mechanical performance, hermeticity, and durability of the devices in both suspended^10,24^ and touch-mode^11^ operation. However, the bending rigidity of the polymer layers reduces the deflection and responsivity of the membranes and sensor. To improve the sensor performance, we therefore gradually remove the transfer polymer by annealing it in dry gas^25–28^ as shown in Fig. 2. PMMA removal mechanisms are mostly related to removing pendant functional groups in the polymer at high temperatures^25,27^. However, this can trigger a reaction between the graphene and the carbon byproducts from the residual polymer and produce amorphous carbon^25,26^. Lower process temperatures and an atmosphere of inert gas, such as argon, are needed to prevent the production of amorphous carbon and to maintain a good quality of graphene during thermal annealing^26^.

The sample is placed inside a furnace and left at a pressure of $500$ Torr with a constant flow of $0.5$ SLPM of an inert dry gas (Ar or N_2_) at a temperature of $300\,^ \circ$C, as schematically depicted in Fig. 2a. We found no notable difference between the use of Ar or N_2_ gas flow in terms of the end quality of the DLG layers or the PMMA removal rate. The average thickness of the transfer PMMA decreases with annealing time at an estimated rate of $\sim \!27$ nm/min, with only minor residues left on and in-between the graphene layers. For some samples, small regions of local PMMA residue accumulation are still present after the annealing procedure. They accumulate on the surface of a DLG layer that has a thickness of $1.4 \!-\! 2.2$ nm, as measured by tapping mode atomic force microscopy (AFM) (see Fig. S1). The PMMA thickness reduction by thermal treatment is found to substantially improve the responsivity of the sensors. Figure 2b shows the capacitance change ${\mathrm{{\Delta}}}C$ of a single chip to external pressure changes between $1000$ and $900$ mbar with a period of 40 s, as measured after $0$, $15$, $22.5$, and $29$ minutes of annealing. Figure 2c shows that by following the thermal annealing procedure, an increase in responsivity of almost an order of magnitude was achieved for this particular sample, while in the best case, an increase in responsivity of nearly two orders of magnitude was observed after a $30$ min continuous thermal annealing treatment. Further annealing of the sample for longer times does not significantly affect the capacitance response (see Fig. S2). The detection noise floor also decreases substantially as a result of the process. The noise floor, ${\mathrm{NF}}$, in Pa/$\sqrt {{\mathrm{Hz}}}$ is defined as:

1 $${\mathrm{NF}} = \frac{{C_{{\mathrm{RMS}}}}}{{\frac{{{\mathrm{d}}C}}{{{\mathrm{d}}P}}\sqrt {f_{{\mathrm{meas}}}} }}$$

where $C_{{\mathrm{RMS}}}$ is the root mean squared (RMS) noise in the capacitance measurement, $\frac{{{\mathrm{d}}C}}{{{\mathrm{d}}P}}$ is the responsivity of the sensor and $f_{{\mathrm{meas}}} = 1.745$ Hz is the frequency at which the measurements are acquired. The decrease in ${\mathrm{NF}}$, as shown in Fig. 2c, qualitatively follows from Eq. (1) considering the measured increase in responsivity.

## Results and discussion

### Sample characterization

**Fig. 3:**
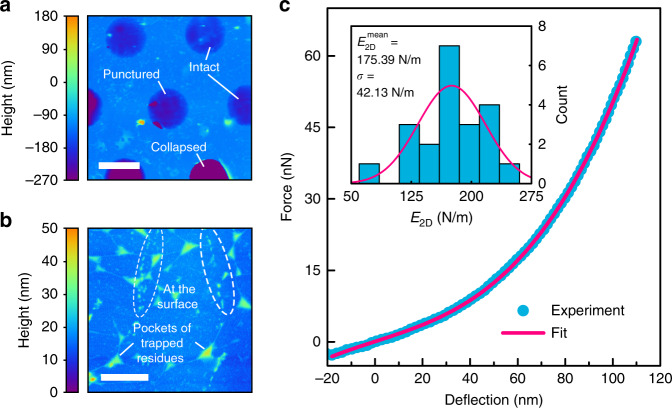
Atomic force microscopy (AFM) characterization of membranes. **a** AFM tapping mode image of DLG drums. The state of the drum is labeled. Scale bar: $5$ $\mu$m. **b** AFM tapping mode image of a supported region of DLG. Residue types are indicated. Scale bar: $1$ $\mu$m. **c** Force versus membrane deflection curve. Experimental data (blue dots) are fit by the membrane model of Eq. (2). Inset: statistics over 21 membranes with the corresponding mean values for the extracted two-dimensional Young’s modulus, $E_{2{\mathrm{D}}}$, with a mean pretension $n_0 = 0.04 \pm 0.02$ N/m

After thermal treatment for 30 min at $300^ \circ$C, we inspect the samples for damage. In Fig. 3a, a tapping mode atomic force microscopy (AFM) image of the sample is shown. Three types of drums can be distinguished visually: intact, ruptured, and collapsed drums. Collapsed drums are in contact with the bottom of the cavity and probably do not significantly contribute to the response^11^ because they are predominantly damaged and thus not airtight. The ruptured drums are also expected to leak fast^17^ and therefore have a negligible contribution to the static capacitance response to gradual pressure changes. Intact drums, however, show full coverage of the cavity. These drums can hermetically seal the cavity with a constant internal pressure, exploiting the extremely low permeability of graphene^2–4^ although in part of these membranes, small pores can be present that are too small to be visually detected. The lower height of some drums visible in Fig. 3a can be caused by the small downward pressure or $P_{{\mathrm{int}}} \,<\, 1$ bar established during the fabrication process. As shown in the AFM measurements in Fig. 3b, after the annealing process, a substantial number of residues are observed on and below the DLG. The residues form pockets and are also observed in the suspended regions of the device, which suggests that parts of the residues are trapped in-between the graphene layers, where they cannot be easily removed^29^.

During fabrication, the capability of graphene to withstand high strains^5,6,30^ facilitates damage-free transfer, while the thickness of only a few atoms favors higher membrane deflections and thus higher responsivity to pressure changes of the sensor. To test the elasticity and mechanical properties of the drums after thermal treatment, we use force-indention AFM to apply a point force at the center of a single membrane while measuring its deflection^5^. The applied force, $F$, is proportional to the stiffness of a cantilever $k_{\mathrm{c}}$ and its deflection ${\mathrm{{\Delta}}}z_{\mathrm{c}}$ as $F = k_{\mathrm{c}}{\mathrm{{\Delta}}}z_{\mathrm{c}}$. We use two cantilevers of $k_{\mathrm{c}} = 1.25 \pm 0.12$ N/m and $1.48 \pm 0.05$ for two separate sets of measurements in two distant places on the chip. We record a force versus membrane deflection curve at the center of each drum, as depicted in Fig. 3c, and fit it to a model of the point deflection of a circular membrane^5,31^:

2 $$F = n_0\pi \delta + E_{2{\mathrm{D}}}Rq^3\left( {\frac{\delta }{R}} \right)^3$$

where $n_0$ is the pretension, $E_{2{\mathrm{D}}}$ is the two-dimensional Young’s modulus of the layer, $\nu = 0.16$ is Poisson’s ratio^7^, $\delta$ is the resulting deflection, $R$ is the radius and $q = 1/\left( {1.05 - 0.15\nu - 0.16\nu ^2} \right)$ is a geometrical factor^5,31^. We use the two-dimensional Young’s modulus, $E_{2{\mathrm{D}}} = E_{3{\mathrm{D}}}h$, for the stacked DLG sheet since the thickness, $h$, of the layer after the thermal treatment is not well defined. This effective quasi-2D Young’s modulus provides a more realistic estimate for the mechanical elasticity of the layer and can be directly compared to that of pristine single-layer graphene^5^. In the inset of Fig. 3c, the statistics over $21$ different drums, which yield a mean value of $E_{2{\mathrm{D}}} = 175$ N/m, are shown. This mean value is substantially lower than the reported values for both exfoliated and pristine CVD single-layer graphene^5,7^ but comparable to those of other CVD graphene membranes^24,30,32,33^, high-quality oxidized graphene sheets^34,35^ and other 2D materials such as single-layer MoS_2_^31^.

**Fig. 4:**
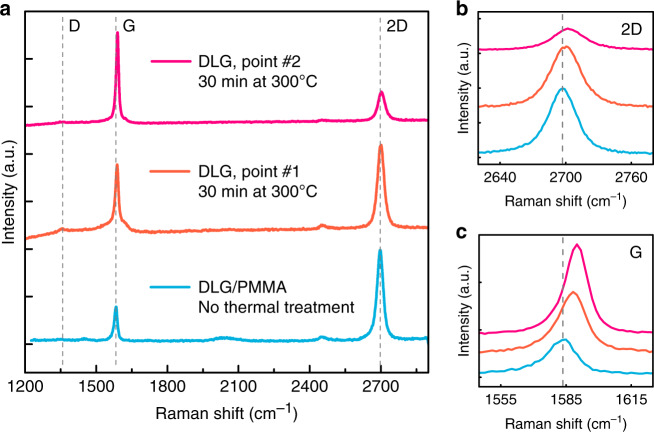
Raman spectroscopy of supported DLG. **a** Raman spectra of the DLG/PMMA layer before and after polymer removal. **b** Close-up of a blueshift in the 2D peak and **c** the G peaks of graphene

We also examined the sheets of DLG by using Raman spectroscopy, as displayed in Fig. 4. Figure 4a shows Raman spectra of DLG acquired before the removal of the PMMA layer (blue line) and after processing at an elevated temperature (orange and magenta line). Before the thermal treatment, the Raman spectrum of the DLG is reasonably homogeneous across the chip, showing a Lorentzian-shaped 2D peak of graphene and a well-defined G peak^36^. The full width at half maximum (FWHM) of the 2D peak is $\sim \!30$ cm^−1^, and the high intensity ratio of 2D to G peaks resembles typical features of pristine graphene^36^. In the case of DLG, this result indicates that the two layers in the stack are well decoupled and/or have, on average, a twist angle^37^ larger than $15^ \circ$. After processing at a high temperature, the width of the 2D peak remains the same (see Fig. 4b), while the ratio of 2D to G peaks changes drastically depending on the chosen location of the measurement on the chip (see Fig. 4a, orange and magenta lines). Additionally, a notable blueshift of both the 2D and G peaks is observed, as shown in Fig. 4b, c. These observations are attributed to a substantial difference in the twist angle across the DLG sheet^37^ as well as local changes in strain as a result of annealing of the graphene layers^38^.

We also note the almost complete absence of the D peak in all Raman spectra, as shown in Fig. 4a, indicating a very low amount of defects in the stacked graphene layers even after exposure to high temperatures^39^. This result is in agreement with the outstanding high-temperature stability of graphene when encapsulated by protective layers^40,41^ and provides evidence that damage caused by the removal of polymer from suspended graphene is minimal^25–28^.

### Analysis of the sensor response

**Fig. 5:**
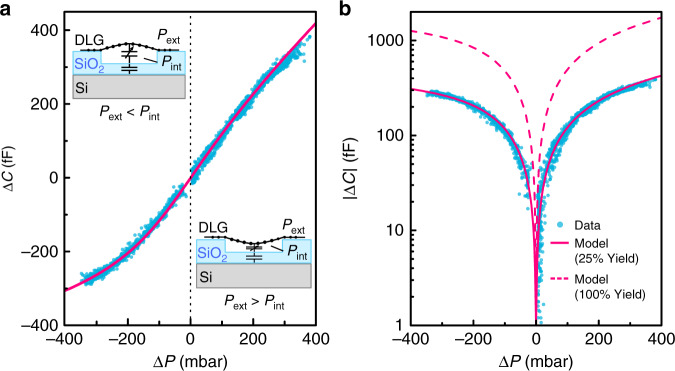
Capacitance-pressure curves of the DLG pressure sensor. **a** Measured capacitance-pressure curve (blue points); solid magenta line is a fit of Eq. (5) to the data, with $P_{{\mathrm{int}}} = 0.933$ bar, $N/N_{{\mathrm{tot}}}$ = 0.25, $E_{2{\mathrm{D}}}$ = 175 N/m and $n_0 = 0.45$ N/m. Insets: schematic images of the effect of membrane deflection. **b** Comparison, on a log-scale, of the measured data (blue points) to the model with $100{\mathrm{\% }}$ (magenta dashed line) and $25{\mathrm{\% }}$ (solid magenta line) yield of the hermetic drums using the 2D Young’s modulus from Fig. 3. All data are acquired during a continuous measurement within a total time of $1$ h at $P_{{\mathrm{ext}}}\, \ne \,P_{{\mathrm{int}}}$ with a maximal rate of change of the gas pressure of $3.3$ mbar s^−1^

A pressure difference ${\mathrm{{\Delta}}}P$ results in a deflection $\delta$ of a circular graphene membrane with radius $R$, given by:

3 $${\mathrm{{\Delta}}}P = \frac{{4n_0}}{{R^2}}\delta + \frac{{8E_{2{\mathrm{D}}}}}{{3R^4(1 - \nu )}}\delta ^3$$

where the graphene membrane takes the shape of the section of a sphere^12,42^. Since the pressure inside the reference cavity is approximately $P_{{\mathrm{int}}} \approx 1$ bar (the pressure during transfer), at $P_{{\mathrm{ext}}} = 1$ bar (atmospheric pressure, ${\mathrm{{\Delta}}}P \approx 0$) and according to Eq. (3), the sensors are expected to have a linear response at small ${\mathrm{{\Delta}}}\delta$. However, at larger deflections, nonlinear mechanical and capacitance effects start to result in nonlinearities in the capacitance $C_{\mathrm{d}}({\mathrm{{\Delta}}}P)$ curve that can be calculated using the parallel-plate approximation^12^ as:

4 $$C_{\mathrm{d}}({\mathrm{{\Delta}}}P) = 2\pi {\it{\epsilon }}_0\mathop {\int }\nolimits_0^R \frac{r}{{g_0 - \delta ({\mathrm{{\Delta}}}P)\left( {1 - \frac{{r^2}}{{R^2}}} \right)}}{\mathrm{d}}r$$

where ${\it{\epsilon }}_0$ is the vacuum permittivity and $g_0$ is the gap size between the membrane and bottom electrode for ${\mathrm{{\Delta}}}P = 0$. The contribution of the quantum capacitance of graphene is small^43,44^ and is neglected. The total capacitance change of the sensor can be modeled from Eqs. (3) and (4) as:

5 $${\mathrm{{\Delta}}}C_{{\mathrm{total}}} = N \times {\mathrm{{\Delta}}}C_{\mathrm{d}}({\mathrm{{\Delta}}}P)$$

where $N$ is the number of intact, hermetic drums after both fabrication and thermal treatment. We experimentally test whether Eqs. (4) and (5) can model the graphene pressure sensor by applying both substantial negative and positive pressure differences while measuring its capacitance. Figure 5a shows the response of the same sensor that was characterized in Figs. 3 and 4.

The maximal responsivity of the sensor is achieved near ambient pressure in the linear regime, while a notable nonlinear response occurs for $|{\mathrm{{\Delta}}}P|\, > \,200$ mbar. A number of design factors, such as the drum diameter, the number of drums and the pitch between the drums, influence the sensor performance^12^. Importantly, the pressure dependence of ${\mathrm{{\Delta}}}C$ can be well reproduced by the model of Eq. (5) using a Young’s modulus $E_{2{\mathrm{D}}} = 175$ N/m, estimated by an AFM probe for this particular sample, and a pretension $n_0 = 0.45$ N/m, as shown by the magenta line in Fig. 5a. The model follows the measurement closely when we use a fitted value of $N/N_{{\mathrm{tot}}}$ = 0.25, as shown in Fig. 5b (solid magenta line), where $N_{{\mathrm{tot}}} \approx 10000$ is the total number of drums. This result indicates that the yield of intact hermetic drums is $25{\mathrm{\% }}$ and suggests that a large number of drums that look visually intact do not remain hermetic after polymer removal. The theoretical maximum response at a perfect yield of $100{\mathrm{\% }}$ is also shown (dashed magenta line).

**Fig. 6:**
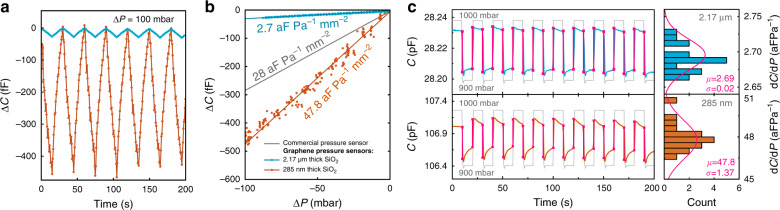
Comparison of different capacitive pressure sensors. **a** Triangular pressure wave applied to two graphene sensors with $285$ nm and $2.17$ $\mu$m oxide thickness, showing the measured change in capacitance. The gas pressure is changed at a rate of $6.25$ mbar s^−1^. **b** Responsivity analysis of the chips at small ${\mathrm{{\Delta}}}P$ using multiple periods of measured data from **a** as a function of the pressure change, and a comparison to a commercial capacitive pressure sensor (Murata SCB10H). **c** Analysis of the responsivity of the chips as determined from periodic pressure steps between $1000$ and $900$ mbar. The gas pressure is changed at a rate of $175.4$ mbar s^−1^. Panels on the left: Blue line—measured capacitance of a graphene sensor with $t_{{\mathrm{SiO}}_2} = 2.17$ $\mu$m. Orange line—measured capacitance of a sensor with $t_{{\mathrm{SiO}}_2} = 285$ nm. Thin gray line—applied time-dependent pressure profile. Magenta lines—extracted capacitance response of the sensors. Panels on the right: statistics over a number of cycles for both oxide thicknesses. The higher responsivity and larger gas leak rate of the $285$ nm device can account for the larger variations. Magenta lines—fit to a normal distribution, with the corresponding mean values $\mu$ and standard deviations $\sigma$ in aF Pa^−1^ indicated

Many other factors can influence the responsivity. Since the device capacitance has a strong dependence on the distance between the plates of the pressure sensor, the cavity depth has a large influence on the performance of the sensor. In Fig. 6, we demonstrate the performance of two of the best samples with SiO_2_ thicknesses of $285$ nm and $2.17$ $\mu$m. Both chips have circular holes with a depth of $240$ nm fabricated as described previously. Figure 6a shows the triangular wave response of both $285$ nm (orange line) and $2.17$ $\mu$m (blue line) chips. As shown in Fig. 6b, the difference in responsivity for the two oxide thicknesses as a function of pressure can be more than an order of magnitude. Using Eq. (1), we calculate the noise floor to be $34.2$ Pa/$\sqrt {{\mathrm{Hz}}}$ for the $285$ nm thick SiO_2_ sample and $43.4$ Pa/$\sqrt {{\mathrm{Hz}}}$ for the $2.17$ $\mu$m sample. The measured responsivity of the $285$ nm gap device is significantly higher than that of the $2.17$ $\mu$m device, in qualitative accordance with Eq. (1).

There is a notable scatter in the measured capacitance values, as seen in Fig. 6b, which is attributed to the effect of gas escaping the cavities for part of the drums, thus causing $|{\mathrm{{\Delta}}}P|$ to decrease with measurement time. It has been reported before that when graphene is suspended over a SiO_2_/Si cavity, it does not always form a perfect hermetic seal^2,3^. As recently shown by Lee, et al., most of the gas permeation in graphene drums occurs along the van der Waals interface between the 2D material and the substrate^2^. As a result of the contribution of this effect, an exponential decrease in $C$ with measurement time is visible in Fig. 6c, in response to periodic pressure steps of ${\mathrm{{\Delta}}}P = 100$ mbar. However, if good graphene crystallinity is preserved over large areas, the mean path that the gas needs to travel to escape the cavities becomes large, which increases the flow resistance of the gas channel and the permeation time constant^2,20^. This condition seems to be achieved across part of the sample because no significant hysteresis was observed during the slow pressure sweeps ($> 10$ minutes per sweep) in Fig. 5a, b, suggesting that on the order of 25% of the drums maintain a constant internal pressure $P_{{\mathrm{int}}}$ for ${\mathrm{{\Delta}}}P\, \ne \,0$, as supported by the fits in Fig. 5a, b.

Optimization of all parameters is required to achieve the best sensor performance for detecting very small pressure differences over an extended range of pressures. DLG pressure sensors show reproducible operation over a pressure range of $65 - 138$ kPa (see Fig. 5a). In terms of responsivity, our best sensor with $285$ nm of SiO_2_ oxide (orange dots in Fig. 6a, b), with a responsivity of $47.8$ aF Pa^−1^ mm^−2^ over the range of $90 - 100$ kPa, already outperforms the commercially available state-of-the-art Murata SCB10H sensor, with a responsivity of $28$ aF Pa^−1^ mm^−2^ in the same range, as shown in Fig. 6b. This outcome is comparably larger than what was previously achieved with capacitive sensors based on atomically thin 2D membranes^9,12^ yet competitive with thicker suspended graphene-covered $140$ nm PMMA sheets^10^, with a reported responsivity of $123$ aF Pa^−1^ mm^−2^ over $0 - 80$ kPa, using an area of $210$ $\mu$m^2^ of tightly packed hexagonal membranes. However, if yields close to $100{\mathrm{\% }}$ and hermetic sealing of all the drums is realized, and if the pretension is decreased to $\sim 0.04$ N/m, the presented graphene-based 2D devices are expected to achieve a theoretical maximum responsivity of $\sim 450$ aF Pa^−1^ mm^−2^ near ambient pressure of $90 - 100$ kPa. Further design improvements might even be possible if the gap is reduced, the unsuspended area is minimized and the packing density of the membranes is increased. Using three or five layers of stacked graphene instead of two might help to improve the hermetic sealing issue but will result in a lower sensitivity (see Fig. S3). Increasing the diameter of drums, on the other hand, could compensate for this and produce an increase in responsivity (see Fig. S4); however, it could also decrease the yield^21^. We demonstrate the proof-of-concept operation of graphene pressure sensors under room temperature conditions ($\sim 23^ \circ$C). Eventually, the temperature-related effects, such as the expansion of gas inside the cavities, small variations in the dielectric constant of the gas as a function of temperature, and thermal expansion of the DLG and Si/SiO_2_ substrate, can potentially affect the capacitance-to-pressure conversion. For operation over an extended temperature range, these need to be excluded by combining the pressure sensor with a temperature sensor^45^, by using appropriate calibration and by sealing the drums under vacuum conditions.

## Conclusions

Where previous studies have addressed some of the challenges related to realizing portable graphene gas pressure sensors, such as the pressure sensitivity, impermeability to gases and electrical readout, here, we bridged these studies by constructing a portable, battery-powered functional graphene pressure sensor that outperforms commercial devices. Using off-the-shelf systems for electronic readout and data processing, we enable capacitive readout of a $1 \times 1$ mm^2^ array of DLG pressure sensors. We realize sensor chips with a high yield of suspended membranes, resulting in a sensor responsivity of $47.8$ aF Pa^−1^ mm^−2^. We demonstrate that thermal treatment is an effective measure for controllable thickness reduction of the support polymer layer, which leads to a significant performance improvement because the thin 2D material membranes are much more flexible than those with the support polymer. It is anticipated that further design and fabrication improvements and better control over the device yield can increase the responsivity by a factor of 10, thus enabling improvements in applications such as indoor navigation, altitude monitoring and activity monitoring, and can enable new applications such as presence detection.

## Supplementary information

SUPPLEMENTARY INFORMATION: Sensitive capacitive pressure sensors based on graphene membrane arrays

## Data Availability

The manuscript has associated data in a data repository. The numerical data shown in figures of the manuscript can be downloaded from the Zenodo online repository at https://doi.org/10.5281/zenodo.4042077.

## References

[1] Zurutuza A, Marinelli C (2014). Challenges and opportunities in graphene commercialization. Nat. Nanotechnol..

[2] Lee M (2019). Sealing graphene nanodrums. Nano Lett..

[3] Bunch JS (2008). Impermeable atomic membranes from graphene sheets. Nano Lett..

[4] Sun PZ (2020). Limits on gas impermeability of graphene. Nature.

[5] Lee C, Wei X, Kysar JW, Hone J (2008). Measurement of the elastic properties and intrinsic strength of monolayer graphene. Science.

[6] Cui T (2020). Fatigue of graphene. Nat. Mater.

[7] Lee G-H (2013). High-strength chemical-vapor-deposited graphene and grain boundaries. Science.

[8] Chen J-H, Jang C, Xiao S, Ishigami M, Fuhrer MS (2008). Intrinsic and extrinsic performance limits of graphene devices on SiO_2_. Nat. Nanotechnol..

[9] Chen Y-M (2016). Ultra-large suspended graphene as a highly elastic membrane for capacitive pressure sensors. Nanoscale.

[10] Berger C, Phillips R, Centeno A, Zurutuza A, Vijayaraghavan A (2017). Capacitive pressure sensing with suspended graphene–polymer heterostructure membranes. Nanoscale.

[11] Berger C (2017). Touch-mode capacitive pressure sensor with graphene-polymer heterostructure membrane. 2D Mater..

[12] Davidovikj D, Scheepers PH, van der Zant HSJ, Steeneken PG (2017). Static capacitive pressure sensing using a single graphene drum. ACS Appl. Mater. Interfaces.

[13] Zhu S-E, Ghatkesar MK, Zhang C, Janssen GCAM (2013). Graphene based piezoresistive pressure sensor. Appl. Phys. Lett..

[14] Smith AD (2013). Electromechanical piezoresistive sensing in suspended graphene membranes. Nano Lett..

[15] Smith AD (2016). Piezoresistive properties of suspended graphene membranes under uniaxial and biaxial strain in nanoelectromechanical pressure sensors. ACS Nano.

[16] Patel RN, Mathew JP, Borah A, Deshmukh MM (2016). Low tension graphene drums for electromechanical pressure sensing. 2D Mater..

[17] Dolleman RJ, Davidovikj D, Cartamil-Bueno SJ, van der Zant HSJ, Steeneken PG (2015). Graphene squeeze-film pressure sensors. Nano Lett..

[18] Romijn, J. et al. A miniaturized low power pirani pressure sensor based on suspended graphene. in *2018 IEEE 13th Annual International Conference on Nano/Micro Engineered and Molecular Systems (NEMS)* (IEEE, 2018).

[19] Rosłoń, I. E. et al. Graphene effusion-based gas sensor (2020). Preprint at https://arxiv.org/abs/2001.09509v1.

[20] Dolleman RJ, Cartamil-Bueno SJ, van der Zant HSJ, Steeneken PG (2016). Graphene gas osmometers. 2D Mater..

[21] Cartamil-Bueno SJ (2017). Very large scale characterization of graphene mechanical devices using a colorimetry technique. Nanoscale.

[22] Barton RA (2011). High, size-dependent quality factor in an array of graphene mechanical resonators. Nano Lett..

[23] Cartamil-Bueno SJ (2016). Colorimetry technique for scalable characterization of suspended graphene. Nano Lett..

[24] Berger CN, Dirschka M, Vijayaraghavan A (2016). Ultra-thin graphene–polymer heterostructure membranes. Nanoscale.

[25] Lin Y-C (2011). Graphene annealing: how clean can it be?. Nano Lett..

[26] Ahn Y, Kim J, Ganorkar S, Kim Y-H, Kim S-I (2016). Thermal annealing of graphene to remove polymer residues. Mater. Express.

[27] Huang L-W (2014). Characterization of the cleaning process on a transferred graphene. J. Vac. Sci. Technol..

[28] Gammelgaard L (2014). Graphene transport properties upon exposure to PMMA processing and heat treatments. 2D Mater..

[29] Jain A (2018). Minimizing residues and strain in 2D materials transferred from PDMS. Nanotechnology.

[30] Ruiz-Vargas CS (2011). Softened elastic response and unzipping in chemical vapor deposition graphene membranes. Nano Lett..

[31] Castellanos-Gomez A (2012). Elastic properties of freely suspended MoS_2_ nanosheets. Adv. Mater..

[32] Nicholl RJ (2015). The effect of intrinsic crumpling on the mechanics of free-standing graphene. Nat. Commun.

[33] Li Z (2015). Deformation of wrinkled graphene. ACS Nano.

[34] Suk JW, Piner RD, An J, Ruoff RS (2010). Mechanical properties of monolayer graphene oxide. ACS Nano.

[35] Gómez-Navarro C, Burghard M, Kern K (2008). Elastic properties of chemically derived single graphene sheets. Nano Lett..

[36] Ferrari AC (2007). Raman spectroscopy of graphene and graphite: Disorder, electron–phonon coupling, doping and nonadiabatic effects. Solid State Commun..

[37] Kim K (2012). Raman spectroscopy study of rotated double-layer graphene: Misorientation-angle dependence of electronic structure. Phys. Rev. Lett.

[38] Ni ZH (2008). Tunable stress and controlled thickness modification in graphene by annealing. ACS Nano.

[39] Eckmann A (2012). Probing the nature of defects in graphene by raman spectroscopy. Nano Lett..

[40] Šiškins M (2019). High-temperature electronic devices enabled by hBN-encapsulated graphene. Appl. Phys. Lett..

[41] Son S-K (2017). Graphene hot-electron light bulb: incandescence from hBN-encapsulated graphene in air. 2D Mater..

[42] Bunch, J. S. *Mechanical and Electrical Properties of Graphene Sheets* (Cornell University Ithaca, NY, 2008).

[43] Ponomarenko LA (2010). Density of states and zero landau level probed through capacitance of graphene. Phys. Rev. Lett..

[44] Xia J, Chen F, Li J, Tao N (2009). Measurement of the quantum capacitance of graphene. Nat. Nanotechnol..

[45] Harzheim A, Könemann F, Gotsmann B, van der Zant HSJ, Gehring P (2020). Single-material graphene thermocouples. Adv. Funct. Mater..

