# Supplementary material for: Sensitive capacitive pressure sensors based on graphene membrane arrays
Source: Microsyst Nanoeng. 2020 Nov 16;6:102. doi: 10.1038/s41378-020-00212-3 (PMC8433463; doi:10.1038/s41378-020-00212-3)
Supplement: Supplementary file 1 — SUPPLEMENTARY INFORMATION: Sensitive capacitive pressure sensors based on graphene membrane arrays [file 41378_2020_212_MOESM1_ESM.docx]

##### SUPPLEMENTARY INFORMATION: Sensitive capacitive pressure sensors based on graphene membrane arrays

Makars Šiškins^1^, Martin Lee^1^, Dominique Wehenkel^2^, Richard van Rijn^2^, Tijmen W. de Jong^1^, Johannes R. Renshof^1^, Berend C. Hopman^1^, Willemijn S. J. M. Peters^1^, Dejan Davidovikj^1^, Herre S. J. van der Zant^1^, Peter G. Steeneken^1, 3^

*^1)^ Kavli Institute of Nanoscience, Delft University of Technology, Lorentzweg 1, 2628 CJ, Delft, The Netherlands*

*^2)^ Applied Nanolayers B.V., Feldmannweg 17, 2628 CT, Delft, The Netherlands*

*^3)^ Department of Precision and Microsystems Engineering, Delft University of Technology, Mekelweg 2, 2628 CD, Delft, The Netherlands*

Correspondence: Makars Šiškins ([m.siskins-1@tudelft.nl](mailto:m.siskins-1@tudelft.nl)), Peter G. Steeneken ([p.g.steeneken@tudelft.nl](mailto:p.g.steeneken@tudelft.nl))

(Dated: 30 September 2020)

**Contents**

**Fig. S1:** Detailed characterization of annealed double-layer graphene in a region where PMMA residues were observed 2

**Fig. S2:** Capacitance response of a DLG sample to pressure step-changes after thermal annealing. 3

**Fig. S3:** Simulated capacitance-pressure curves of the graphene pressure sensor with a different number of layers and pre-tension. 3

**Fig. S4:** Simulated capacitance-pressure curves of the graphene pressure sensor with a different diameter of drums and pre-tension. 4


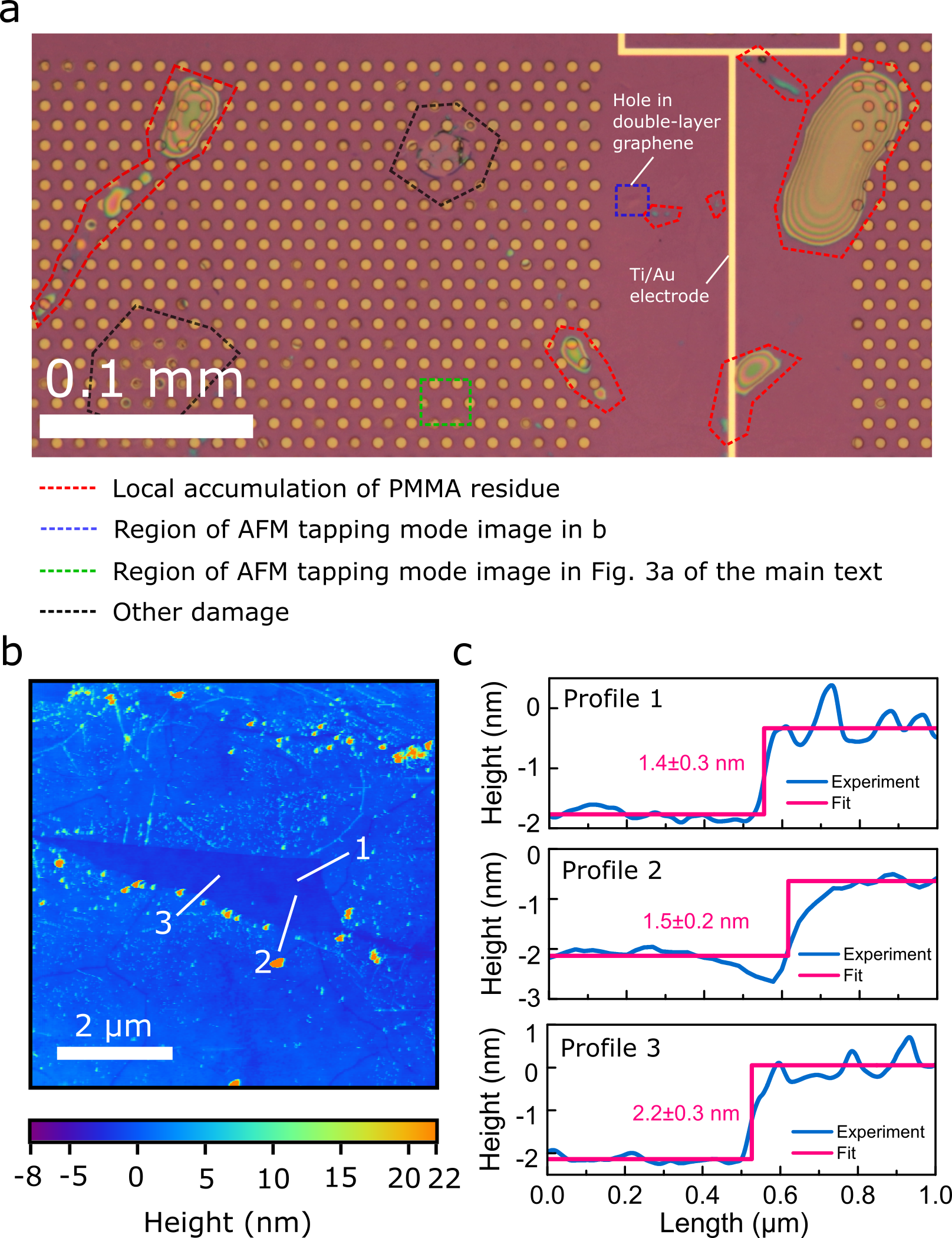


FIG. S1. Detailed characterization of annealed double-layer graphene in a region where PMMA residues were observed. (a) Optical image of a thermally annealed graphene sample with a $285$ nm thickness of SiO_2_, results of which are also presented in Fig. 3 of the main text. (b) Tapping mode AFM image of the area indicated with a dashed blue line in (a). Numbered white lines indicate corresponding height profiles in (c). The extracted thickness of DLG is indicated for each case.


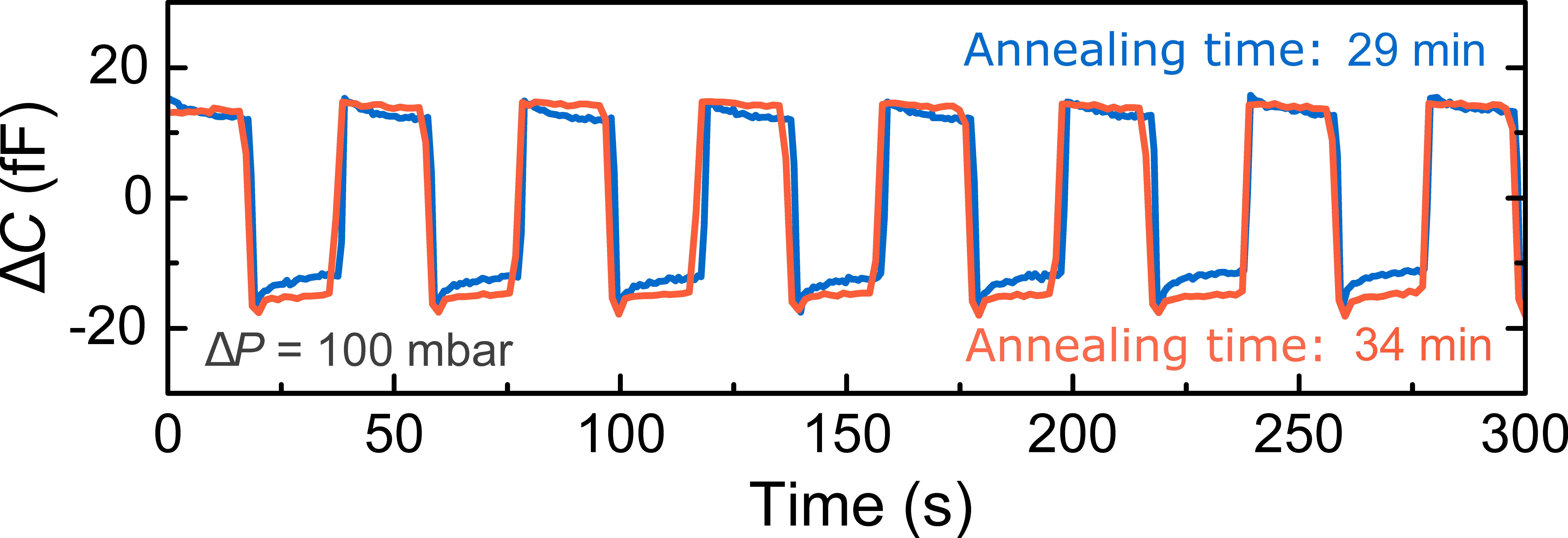


FIG. S2. Capacitance response of a DLG sample to pressure step-changes after thermal annealing. Coloured lines - change in capacitance of a single chip as a function of measurement time in response to $\Delta P$, annealed for a time indicated in the legend.


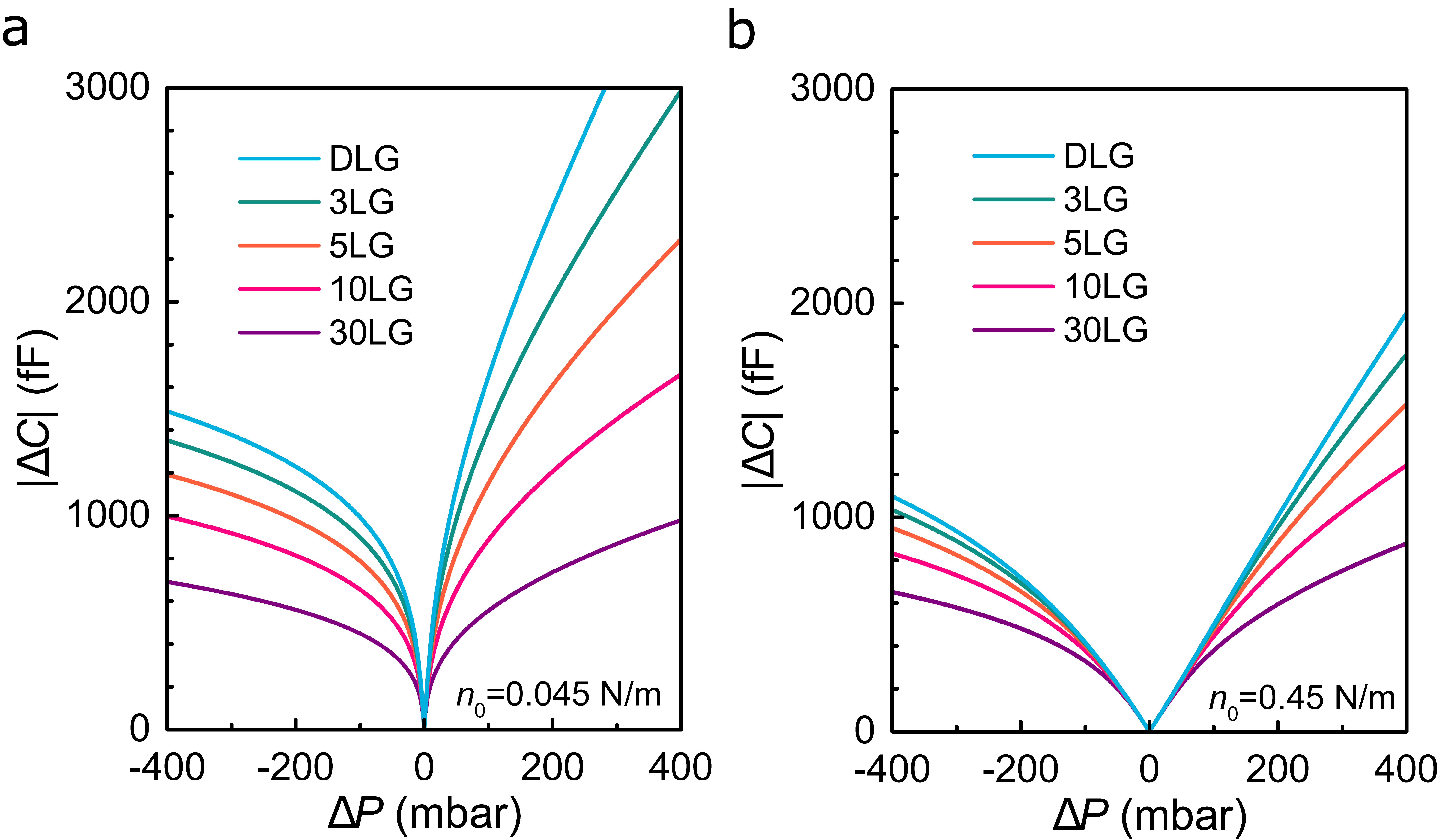


FIG. S3. Simulated capacitance-pressure curves of the graphene pressure sensor with a different number of layers and pre-tension of (a) 0.045 N/m and (b) 0.45 N/m.


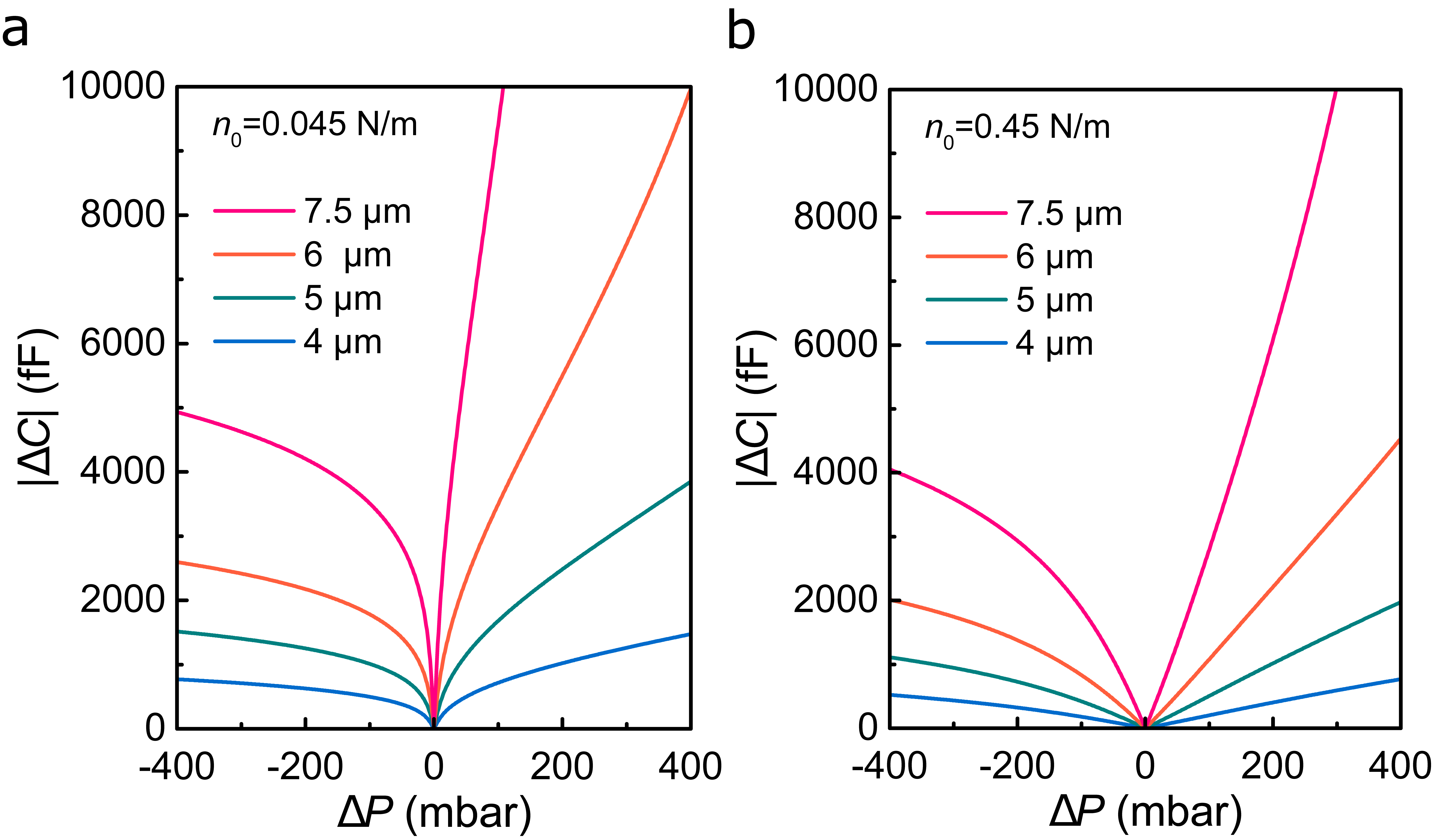


FIG. S4. Simulated capacitance-pressure curves of the graphene pressure sensor with a different diameter of drums and pre-tension of (a) 0.045 N/m and (b) 0.45 N/m.
